# Acute Secondary Prevention of Ischemic Stroke: Overlooked No Longer

**DOI:** 10.3389/fneur.2021.701168

**Published:** 2021-09-10

**Authors:** Zachary B. Bulwa, Scott J. Mendelson, James R. Brorson

**Affiliations:** ^1^Department of Neurology, Rush University Medical Center, Chicago, IL, United States; ^2^Department of Neurology, The University of Chicago, Chicago, IL, United States

**Keywords:** acute stroke, ischemic stroke, antiplatelet, statin therapy, timing of intervention, anticoagulation timing

## Abstract

Recent studies of interventions initiated acutely following onset of minor ischemic stroke or transient ischemic attack (TIA) have disclosed early stroke recurrence rates that are substantially higher than long-term recurrence rates and that can be reduced by acute antiplatelet treatment interventions. These observations, bolstered by analysis based on kinetic modeling of the time course of recurrence following initial events, suggest that acute stroke patients experience an underlying vulnerable state that quickly transitions to a more stable state. Some evidence also supports the benefits of early treatment with direct-acting oral anticoagulants in cardioembolic stroke and of continuation or early initiation of statin therapy in atherosclerotic stroke. Treatment of ischemic stroke should address the transient vulnerable state that follows the initial event, employing measures aiming to avert early recurrence of thromboembolism and to promote stabilization of vulnerable arterial plaque. These measures constitute *acute secondary prevention* following ischemic stroke.

## Introduction

In the hospital care of acute ischemic stroke patients, initial clinical management is tightly focused on interventions aimed to reverse ischemia through induced reperfusion and to limit early complications of brain infarction. Investigations are undertaken to rapidly explore potential mechanisms of stroke, and planning for discharge quickly begins, with selection of the appropriate rehabilitation program to meet the patient's needs. Careful consideration of preventing a subsequent stroke is often relegated to the ambulatory setting. Instead, secondary prevention needs to be considered acutely, as the highest risk for recurrent stroke is typically in the first several days following an initial ischemic event (1, 2). Risks of early recurrence depend on the subtype of ischemic stroke and on individual patient features, and acute interventions to prevent recurrence need to be targeted to the specifics of each case.

## Antiplatelet Treatment in Minor Stroke or Transient Ischemic Attack

Early secondary stroke prevention trials generally had enrollment windows extended for months following the ictus, missing early detection of stroke recurrence. Exceptions were the Chinese Acute Stroke Trial (3) and the International Stroke Trial (4), which examined early initiation of aspirin within 48 h following ischemic stroke, demonstrating modestly high early recurrence rates that were reduced by aspirin. More recently, trials of acute treatment of minor ischemic stroke or TIA with augmented antiplatelet regimens, such as CHANCE (Clopidogrel in High-Risk Patients with Acute Non-disabling Cerebrovascular Events) (5), SOCRATES (Acute Stroke Or Transient IsChaemic Attack TReated With Aspirin or Ticagrelor and Patient OutcomES) (6), POINT (Platelet-Oriented Inhibition in New TIA and Minor Ischemic Stroke) (7), and THALES (Transient IscHaemic Attack Treated With TicAgreLor and ASA for PrEvention of Stroke and Death) (8), with randomization and tracking of subjects occurring within 12–24 h of stroke onset, have demonstrated a clear and consistent finding: the stroke recurrence rate is highest within the first few days following stroke, slowing to a second phase of lower recurrence rate, sustained over subsequent months. In each of these trials of acute antiplatelet regimens, the majority of outcome events, predominantly consisting of ischemic strokes, occurred within the first 7 days, in both control and active treatment groups (see Table 1). Further, the effects of dual antiplatelet therapy with aspirin and clopidogrel (in CHANCE and POINT) or with ticagrelor (in THALES) over aspirin monotherapy appeared to be confined to a reduction of the rate of early recurrence, with plots of subsequent survival free of stroke after the first few weeks running in parallel in dual antiplatelet therapy and aspirin monotherapy groups.

**Table 1:** Evidence for front-loading of stroke recurrence in selected stroke treatment trials, based on intention-to-treat data for the primary trial outcome event definition (MI: myocardial infarction).

**Trial**	***N***	**Treatments (following load)**	**Event rates**			**Fraction in 7 days**	**Hemorrhage rates**
			**7 days**	**30 days**	**90 days**		**90 days**
CHANCEPrimary outcome: any stroke	2,586	Aspirin 75 mg QD	8.7%	10.2%	11.7%	0.74	0.3%
	2,584	Clopidogrel 75 mg plus aspirin 75 mg QD × 21 days	5.9%	7.3%	8.2%	0.72	0.3%
SOCRATESPrimary outcome: stroke, MI, or death	6,610	Aspirin 100 QD	4.9%	5.8%	7.5%	0.65	0.6%
	6,589	Ticagrelor 90 BID	3.9%	5.2%	6.7%	0.58	0.5%
POINTPrimary outcome: stroke, MI, or vascular death	2,449	Aspirin 50–325 mg QD	4.6%	5.9%	6.5%	0.71	0.4%
	2,432	Clopidogrel 75 mg QD plus aspirin	2.9%	4.1%	5.0%	0.58	0.9%
THALESPrimary outcome: stroke or death	5,493	Aspirin 75–100 mg QD	5.3%	6.6%	(–)	0.80	(0.1% at 30 days)
	5,523	Ticagrelor 90 mg BID plus aspirin	4.2%	5.5%	(–)	0.76	(0.5% at 30 days)
Yaghi et al. (23) (stroke in atrial fibrillation; observational) Primary outcome: stroke, TIA, or arterial embolism	862	DOACs	1.7%	3.1%	4.2%	0.40	
	389	Warfarin	3.3%	5.3%	8.0%	0.41	

*Approximate event recurrence rates are derived from published graphical data and are used to estimate the fraction of events over the entire 90- or 30-day trial duration that occurred within the first 7 days. In each of these studies, ischemic stroke constituted the majority of outcome events*.

Analyses of the CHANCE and POINT trials have confirmed that the benefits of dual antiplatelet therapy over aspirin alone accrued entirely in the first few weeks following stroke, while the small excess bleeding risk accompanying dual antiplatelet therapy continued at an approximately constant rate over the entire duration of the study period (9, 10). For these reasons, several authors have recommended adoption of a dual antiplatelet therapy regimen modified from that of the POINT trial, extending for only a 3-week period rather than 3 months, to gain the early benefit without the extended increased hemorrhage risk.

The aforementioned antiplatelet trials were selected for patients with minor stroke or TIA, arguably enriching strokes of atherosclerotic origin by excluding strokes due to cardioembolism. A *post-hoc* analysis of the CHANCE trial data found that the risk of recurrent stroke was substantially greater in those patients with intracranial atherosclerosis as the probable mechanism of stroke and that risk reductions of dual antiplatelet therapy were numerically confined to this group, though the interaction of subgroup with treatment effect did not reach significance (11). Furthermore, pre-specified analyses of the SOCRATES and THALES trials showed a superiority of ticagrelor (alone or added) over aspirin alone for prevention of stroke recurrence that was confined to the subgroup of subjects with identified ipsilateral atherosclerotic disease (12, 13). A plausible interpretation is that embolic events due to unstable or ulcerated atherosclerotic arterial plaques are particularly associated with high rates of recurrent embolism for a brief period, perhaps until ulceration heals or intraplaque inflammation quiets, explaining both the transient high rate of early stroke recurrence and the efficacy of augmented antiplatelet therapy.

**Figure 1:**
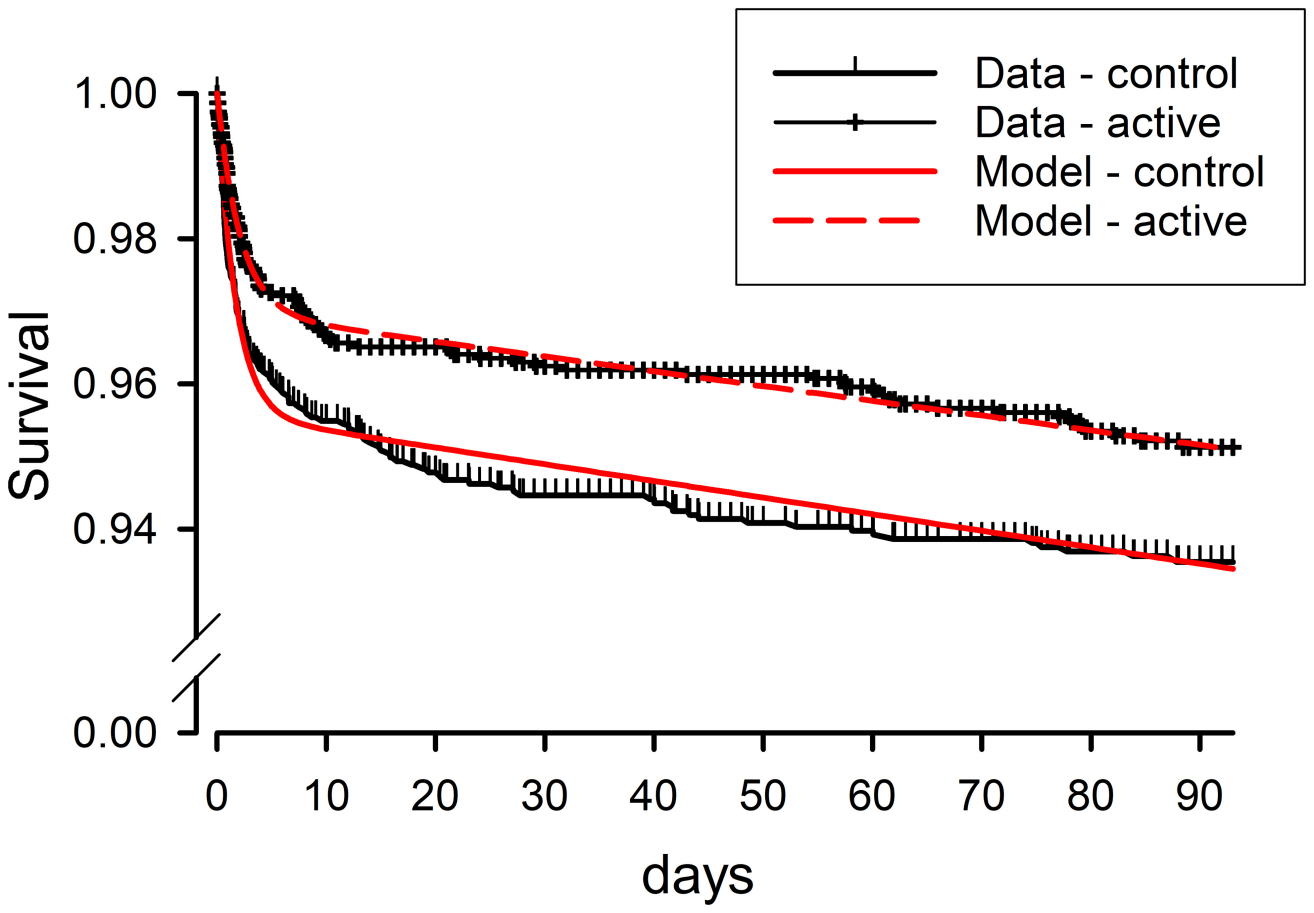
Clinical survivor function data from the POINT trial for active treatment (clopidogrel + aspirin) and control (aspirin) as-treated groups, compared to results predicted by fitting of these data by a mathematical model based on kinetic analysis. Survival free from the composite primary endpoint of ischemic stroke, myocardial infarction, or death is plotted vs. time from study entry, within 12 h of the initial minor stroke or TIA. The kinetic model postulates two clinical states for patients following initial stroke or TIA, one state vulnerable to a high rate of recurrence of ischemic events, but short-lived, and the other state stabilized, with ischemic events recurring at a low rate. Modified from Brorson and Bulwa (14).

While the front-loading of stroke recurrence demonstrated in these trials has been repeatedly recognized, there has been less explicit recognition of a clear implication: that subjects in the acute stroke prevention trials must be distributed between more than one clinical state to produce this sort of temporal pattern. If all subjects were in a single state with constant risks of stroke recurrence, the kinetics of survival free of stroke recurrence would follow a simple exponential decline. Instead, the two phases of recurrence rates seen in trial outcome data require a two-state kinetic model, postulating a transient vulnerable state and a long-term stabilized state. Such a model produces a close mathematical match to the temporal kinetics of Kaplan–Meier curves from the POINT trial (Figure 1). It also gives estimates for the kinetic rates of event recurrence in the vulnerable and stabilized states and for the rate of transition from the vulnerable to stabilized state (14). Notably, the predicted kinetic rates for stroke recurrence in the vulnerable state are ~100-fold greater than the rates in the stabilized state, underscoring the imperative to direct acute preventative treatment to the mechanisms producing the vulnerable state.

## Anticoagulation in Cardioembolic Stroke

The following question then arises: whether in cardioembolic stroke there is a similar two-phase time course of stroke recurrence, with a high early rate followed by a constant lower rate over subsequent months and years. Older trials of anticoagulation for atrial fibrillation or cardiomyopathy did not selectively enroll patients in the immediate period following stroke, missing any opportunity to detect an early high-risk phase of stroke recurrence (15–17). A large single-center case series showed a substantial rate of 3.9% of recurrent embolism within 7 days in cardioembolic stroke patients strongly predicting in-hospital mortality (18). Thus, while data are limited regarding early recurrence rates following cardioembolic strokes, there may be reason for urgency in starting anticoagulation therapy for secondary prevention. Current guidelines only recommend that in the setting of atrial fibrillation, it is reasonable to start anticoagulation within 4–14 days of the onset of symptoms (19), but it is also reasonable to delay anticoagulation past 14 days in patients with higher risks (20).

The optimal timing of anticoagulation with direct-acting oral anticoagulants (DOACs) following ischemic stroke in atrial fibrillation is the focus of ongoing trials. Existing evidence shows that anticoagulation with DOACs in the early period (3–5 days) following ischemic stroke at least can be applied with a low frequency of associated symptomatic intracranial hemorrhage events (21, 22). Analysis of multicenter observational data comparing different strategies for anticoagulation following atrial fibrillation-associated stroke supports the use of DOACs over warfarin and supports direct initiation of oral treatment rather than bridging with heparin or heparinoids (23). These data show a noticeable front-loading of recurrent ischemic stroke, especially in the warfarin-treated patients, with events occurring in ~3% of subjects in the first 7 days after stroke, compared to 8% at 90 days (see Table 1). Bleeding events occurred predominantly with bridging with heparin or heparinoids in the first weeks following stroke, with a 3.1% bleeding risk within 30 days. Thus, the currently available data suggest an elevation of early embolism recurrence after atrial fibrillation-associated ischemic stroke, which may be safely countered by early initiation of direct oral anticoagulants, without heparin bridging. Further studies are in progress aiming to better define the optimal timing of initiation of anticoagulation (21).

## Cervical Carotid Artery Disease Management

Cervical carotid artery disease raises an additional dilemma regarding acute secondary prevention: unstable plaque in the internal carotid artery is well-recognized to lead to a very high risk for recurrent stroke, particularly in the early period following initial stroke or TIA (24), and yet risks of hemorrhagic transformation of recent infarction raise concerns regarding early carotid revascularization with endarterectomy or stenting procedures. At one time, surgeons approached acutely symptomatic carotid stenoses with caution, frequently delaying surgery for 3 weeks or more, theoretically allowing restoration of cerebrovascular reactivity in the recently ischemic tissue before revascularizing the symptomatic carotid. However, secondary analysis of the European Carotid Surgery Trial and North American Symptomatic Carotid Endarterectomy Trial data for effects of timing of surgery following symptoms showed that in patients with symptomatic stenosis, risk reductions provided by surgery fell substantially when surgery was delayed beyond 2 weeks (24). Risks of surgery are no higher in neurologically stable patients with recent TIA or nondisabling stroke when operated on early, within the first 1 week from the event, as compared to those undergoing endarterectomy later (25). American Heart Association guidelines recommend that it is reasonable to perform carotid revascularization, when indicated, within 2 weeks of a TIA or minor nondisabling stroke (20).

A subgroup of cervical carotid stroke patients has a distinctive pattern of crescendo TIA or stroke-in-evolution, often due to hypoperfusion resulting from severe carotid stenosis, with progressive symptoms unresponsive to medical stabilization, and a high risk of severe stroke outcome. These observations have driven a trend toward earlier carotid revascularization in selected cases of unstable ischemia, with acceptable complication risks reported in a large observational study of carotid endarterectomy performed within 48 h of onset of TIA or stroke-in-evolution (26). However, a systemic review of published studies indicates that early intervention in such cases clearly comes at the cost of higher absolute risks of stroke and death (25). Risks and potential benefits of carotid intervention depend on the particular clinical context, including clinical or radiographic evidence for unstable plaque or hypoperfusion due to occlusion, and therefore, decisions regarding the performance and timing of revascularization need to be individualized for each patient.

## Cryptogenic Stroke

One-quarter to one-third of ischemic strokes remain unexplained after standard inpatient etiologic evaluations. Many of these cases have features strongly pointing to an embolic mechanism of stroke. These cases have been categorized as “Embolic stroke of undetermined source,” or ESUS, and the supposition that many of these events are occurring due to cardioembolism has led to testing of anticoagulation as a potentially more efficacious method of secondary prevention than aspirin. Thus far, randomized trials have not shown any superiority of DOACs over aspirin for secondary prevention following ESUS (27, 28). These trials had long windows of enrollment following the initial stroke of up to 6 months, and thus, they do not provide insight into the rate of early recurrence of stroke. Further study is needed to determine if active short-lived mechanisms producing the initial stroke in ESUS may also contribute to increased rates of early stroke recurrence in this setting.

## Effects of Lipid-Lowering Therapy

Though the role of statins in long-term secondary prevention in stroke of presumed atherosclerotic mechanism is well-established, data regarding early initiation of statin therapy are limited. A large retrospective study showed that patients on statins prior to stroke hospitalization had improved post-stroke survival, especially when statins were restarted with 2 days of the stroke, whereas statin withdrawal at the time of the stroke was associated with increased mortality (29). A small randomized trial comparing a 3-day interruption in statin treatment to statin continuation at the time of stroke showed higher rates of early neurological deterioration and of dependency at 3 months with statin withdrawal (30). Two small randomized trials have attempted to test the effect of early vs. late initiation of statin therapy after stroke. Starting atorvastatin 80 mg at day 3 vs. at day 30 made no significant difference in the growth of infarction volume (31). Various statins started within 24 h vs. after 7 days following stroke in the ASSORT (Administration of Statin on Acute Ischemic Stroke Patient) trial did not significantly affect disability at 90 days or the rate of ischemic stroke recurrence (32). Thus, prospective trials have not yet defined the best timing for initiation of statins following stroke. Despite the lack of definitive evidence, existing data suggest that statin therapy should not be withdrawn at the time of stroke. Instead, in patients previously on treatment, statins should be continued at the time of stroke, and in patients with appropriate indications not previously treated with statins, they should be initiated within 1 or 2 days of the stroke.

## Discussion

Driven by recent clinical trials examining the early hours following the initial stroke, management of acute stroke and TIA has begun to address *acute secondary prevention*. Clear evidence has established the efficacy of dual antiplatelet therapy with clopidogrel and aspirin and has suggested a possible role for ticagrelor. In atrial fibrillation-related stroke, early institution of anticoagulant therapy with DOACs may also safely prevent an initial wave of stroke recurrence. Statin treatment during and after stroke admission is associated with lowered mortality and dependency following the stroke and is generally indicated for long-term secondary prevention. Further investigations will need to explore ways to promote plaque stabilization following initial atheroembolic events from ruptured plaque, perhaps the chief entity accounting for the vulnerable state transiently following an initial ischemic event. Efforts aimed at preventing early stroke recurrence have long-term consequences for patients, including averting cognitive impairment, a frequent consequence of incident and recurrent lacunar stroke (33, 34). Hospital care for acute stroke patients must begin to emphasize evidence-based *acute secondary prevention* in the transition between acute treatments and long-term preventative care.

## Author Contributions

ZB and JB developed the concept for this article. JB wrote the first draft and edited further drafts. ZB and SM reviewed and revised the article. All authors agree to be accountable for the content of the work.

## Funding

This work was internally funded through academic support of the Department of Neurology of the University of Chicago.

## Conflict of Interest

The authors declare that the research was conducted in the absence of any commercial or financial relationships that could be construed as a potential conflict of interest.

## Publisher's Note

All claims expressed in this article are solely those of the authors and do not necessarily represent those of their affiliated organizations, or those of the publisher, the editors and the reviewers. Any product that may be evaluated in this article, or claim that may be made by its manufacturer, is not guaranteed or endorsed by the publisher.
